# Influence of Interspecific Competition and Landscape Structure on Spatial Homogenization of Avian Assemblages

**DOI:** 10.1371/journal.pone.0065299

**Published:** 2013-05-28

**Authors:** Oliver J. Robertson, Clive McAlpine, Alan House, Martine Maron

**Affiliations:** 1 School of Geography, Planning and Environmental Management, The University of Queensland, St Lucia, Queensland, Australia; 2 Ecosure, West Burleigh, Queensland, Australia; The Australian National University, Australia

## Abstract

Human-induced biotic homogenization resulting from landscape change and increased competition from widespread generalists or ‘winners’, is widely recognized as a global threat to biodiversity. However, it remains unclear what aspects of landscape structure influence homogenization. This paper tests the importance of interspecific competition and landscape structure, for the spatial homogeneity of avian assemblages within a fragmented agricultural landscape of eastern Australia. We used field observations of the density of 128 diurnal bird species to calculate taxonomic and functional similarity among assemblages. We then examined whether taxonomic and functional similarity varied with patch type, the extent of woodland habitat, land-use intensity, habitat subdivision, and the presence of *Manorina* colonies (a competitive genus of honeyeaters). We found the presence of a *Manorina* colony was the most significant factor positively influencing both taxonomic and functional similarity of bird assemblages. Competition from members of this widespread genus of native honeyeater, rather than landscape structure, was the main cause of both taxonomic and functional homogenization. These species have not recently expanded their range, but rather have increased in density in response to agricultural landscape change. The negative impacts of *Manorina* honeyeaters on assemblage similarity were most pronounced in landscapes of moderate land-use intensity. We conclude that in these human-modified landscapes, increased competition from dominant native species, or ‘winners’, can result in homogeneous avian assemblages and the loss of specialist species. These interacting processes make biotic homogenization resulting from land-use change a global threat to biodiversity in modified agro-ecosystems.

## Introduction

The global biosphere is currently undergoing a decline in the distinctiveness of local and regional biotic assemblages, and represents a serious challenge for conservation biogeography [1]. McKinney and Lockwood [1] coined the term biotic homogenization as the process that replaces unique endemic species with already widespread species. Later it was more specifically defined as a process where range-expanding habitat generalists invade new species pools at the expense of rare or endemic specialist species that disappear [2]. Taxonomic homogenization refers to the increasing similarity of species assemblages across time and space, whilst functional homogenization refers to the increasing similarity of functional ‘roles’ within communities [3]. More broadly, biotic homogenization has been attributed to the increasing dominance of generalist species [1], [4], resulting in a loss of beta (β) diversity, often with a concurrent decline in local or alpha (α) diversity [3]. Several studies have predicted a mass extinction of more than 50% of the world’s species, during a Homogocene [5], wherein distinct communities are replaced by cosmopolitan communities [1], [6].

Biotic homogenization was initially attributed to invasion by non-native species in response to the globalization of commerce and transport [7], [8]. More recently, renewed interest in biotic homogenization has revealed non-random impacts of anthropogenic activities, including urbanization, atmospheric pollution and agricultural land-use, on local native species assemblages [9]. Geographic range expansion of widespread generalist species is occurring at the expense of sensitive specialist species [2]. The so called ‘winners’ of biotic homogenization often share similar traits such as high fecundity, rapid dispersal, broad diets and a tolerance of human disturbance, whilst in contrast the ‘losers’ of biotic homogenization often require specific habitat types and low levels of landscape modification [1]. Biotic homogenization is often driven by novel disturbance regimes and permanent changes to landscape structure [4], [10]. For example, frequent forest-cutting and fire in the central New England Region of the United States during the early 17^th^ century, presented a novel and massive disturbance regime which disrupted forest dynamics at a regional scale with long-term changes to floristic composition and similarity [11]. More recently, Ekroos *et al.* [12] demonstrated land-use induced biotic homogenization of *lepidopteran* communities in Finnish agricultural landscapes as a result of landscape modification. This study showed a decrease in beta-diversity in response to increasing arable field cover at the landscape scale, associated with an increase in the proportion of generalists and highly mobile butterfly species.

Increased competition from successful generalist species may also enhance the process of biotic homogenization in human-modified landscapes, although this hypothesis remains untested. In Australia, cooperative interspecific aggression by the noisy miner (*Manorina melanocephala*), a native honeyeater, is known to have a strong impact on the structure of avian species assemblages across the agricultural and woodland landscapes of eastern Australia [13]. These cooperative breeders form large colonies with all individuals contributing to territory defense. A congener, the yellow-throated miner (*M. flavigula*), may have a similar influence on assemblage structure [14]. The noisy miner is well known for reducing species richness of woodland bird communities and excluding smaller species [15]–[18]. Therefore, in Australian agricultural regions, both landscape change and altered interspecific interactions may act synergistically as drivers of biotic homogenization.

In this paper, we addressed the question: does landscape structure and competition from a widespread generalist native species, drive taxonomic and functional homogenization across space? We defined spatial biotic homogenization as an increase in the similarity of assemblage composition through space (i.e. among sites). Specifically, we defined taxonomic homogenization as an increase in similarity based on species composition, and functional homogenization as an increase in similarity based on functional group composition. We tested whether woodland patch type, habitat extent, woodland habitat subdivision, land-use intensity and interspecific competition affected biotic homogenization of woodland bird communities in a fragmented agricultural landscape. We also tested the relationship between the mean degree of specialization of avian assemblages and landscape structure and interspecific competition.

## Methods

### Study area and survey sites

**Figure 1:**
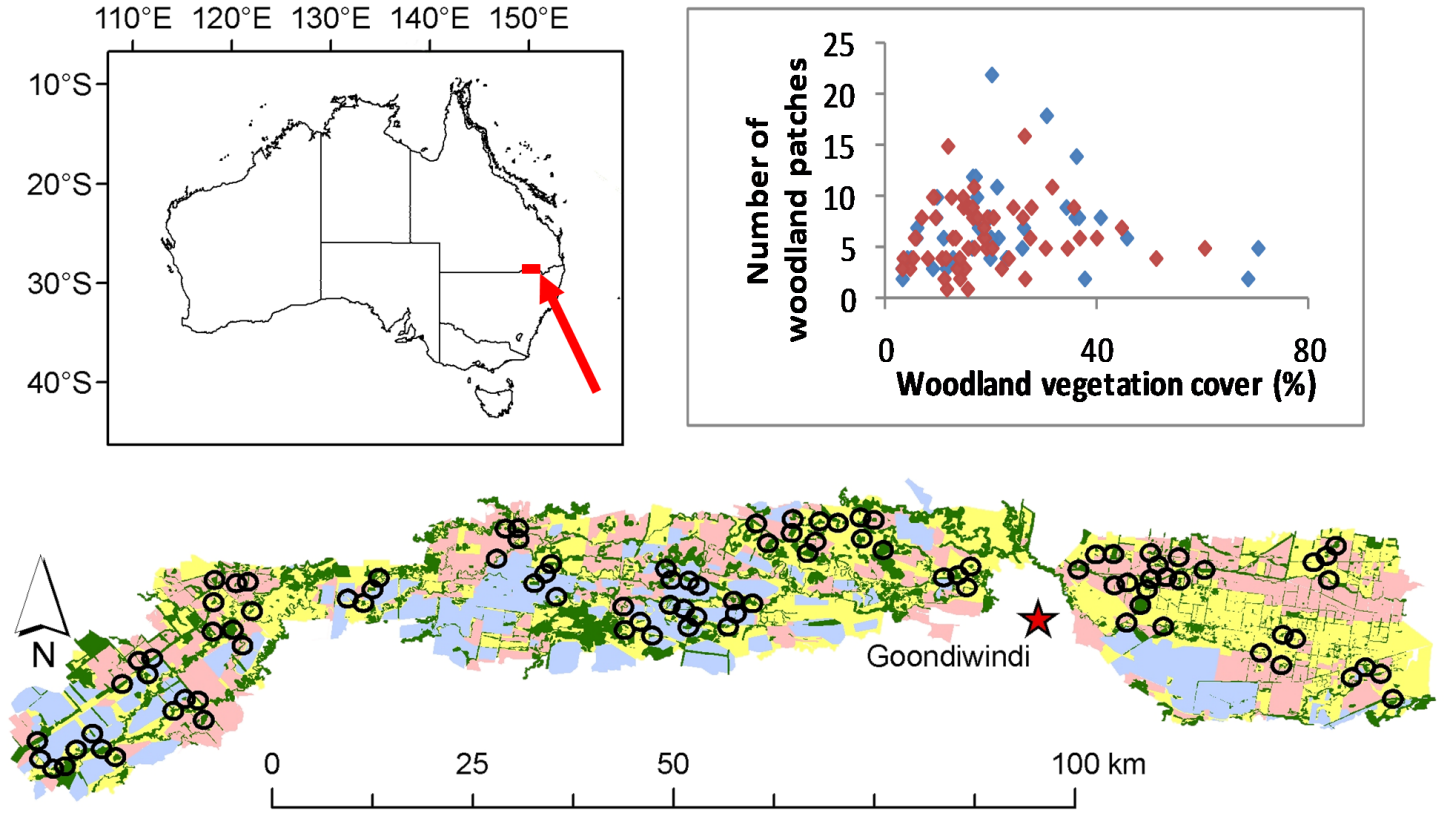
Location of the study area in southern Queensland (red arrow), and location of the 96 study landscapes (1 km radius) in relation to the nearest township of Goondiwindi, as indicated by a star, and the Queensland border along the Macintyre River. Green shading represents woodland vegetation, irrigated land-use shown in blue, dryland land-use shown in pink and pastoral land-use shown in yellow. Also a scatter plot displaying the relationship between the extent and subdivision of woodland habitat within study landscapes, with sites colonized by *Manorina* honeyeaters shown in red and sites where colonies are absent shown in blue.

The study was conducted within the Border Rivers Catchment on the Queensland side of the Macintyre River in southern Queensland, Australia (Figure 1). Major vegetation types include *Eucalyptus* open/grassy woodlands and *Casuarina* woodlands, with river red gum (*E. camaldulensis*) open forests dominating riparian areas. Land clearance for cropping (cotton and cereal) and pastures began in the 1950s [19], and continued until 2004, when state legislation was introduced to control broad-scale clearing. The current extent of native vegetation in the study area is 17%, with 22% of the region used for irrigated cropping, 27% for dryland cropping and 34% for cattle and sheep pastures. Native woodland ecosystems are highly fragmented with a mean patch size of 15 ha (SD  = 37); however, woodlands have high structural connectivity with a linear network of woodland strips and riparian woodlands.

We selected a grid of 5×5 km squares using ArcMap 9.3. Twenty-four squares were subjectively selected to cover a range of landscape patterns. Four 2 ha (50×400 m) survey sites were selected within each grid square to minimize logistic requirements including transport and property access rights. Sites were distributed across four different woodland patch types to minimize bias towards certain patch types. For each grid square, one site was positioned at the centre of a large woodland patch (> 30 ha), a small woodland patch (≤30 ha), within a riparian woodland patch (woodland fringing creeks and drainage lines), and within a linear woodland patch (roadside or fence line vegetation). Sites were separated by a minimum of 1 km.

### Bird surveys

Within each study site, the observed density of all diurnal birds was recorded for 20 minutes using the active search method. This method allows observers to flush and identify cryptic or quiet species within the search area to make certain of identification, with counts of birds during a specified time period providing an index of abundance [20]. A single observer (O.R.) walked in a zigzag pattern along the length of the survey area, covering the entire area without back-tracking unless new species appeared, identifying birds to species level by sight and/or sound. Birds above the canopy were not recorded with the exception of aerial insectivores, predators and scavengers. Surveys were conducted up to 4 hours after sunrise and 2 hours before sunset. Nine repeat surveys were conducted on non-consecutive days for each site between March 2009 and May 2010. Survey effort was equally distributed across three seasons, with 3 repeats per season: autumn 2009, spring 2009 and autumn 2010.

### Assemblage similarity

We pooled density data from the three seasons into a single data frame to increase the detection of nomadic species within survey sites, which move over large distances and appear more sporadically at sites across the landscape, compared to resident species. Few seasonal migrants occurred in the assemblages, which showed little compositional variation between seasons.

To quantify taxonomic similarity, we analyzed a Bray-Curtis matrix based on summed counts of all 128 species (*Manorina* honeyeaters excluded) across all 96 sites. We utilized a log(x+1) data transformation to reduce the dominating influence of abundant taxa and increase the influence of rare species. To quantify functional homogenization, we analyzed a Bray-Curtis matrix based on summed counts of all members of functional groups (except *Manorina* honeyeaters) across all 96 sites. Functional groups were defined *a priori* and based on primary diet and primary foraging strata, resulting in 15 unique groups (Table 1). We also used log(x+1) transformations for the density estimate of each functional group.

**Table 1:** The number of species within each of the 15 functional groups defined by both diet and foraging strata, used in the analysis of functional homogenization.

	Foraging strata				
Primary diet	Ground	Shrub	Branch	Canopy	Aerial
Granivores	26	0	–	1	–
Frugivores	1	1	–	4	–
Insectivores	29	11	2	15	13
Nectarivores	–	3	–	13	–
Carnivores	9	0	0	1	9

By using this method species with the same diet but different foraging strata were in different groups and vice versa. Each cell represents a unique functional ‘role’ or niche. Blank (–) cells represent niches with no members, whilst cells with a value of ‘0’ represent niches unfilled by the local avian assemblage.

### Habitat specialists

We calculated a Specialization Index (SI) for all species detected in the study area by counting the number of main habitat associations of each species from a list of 24 potential habitat categories ranging from rainforest to grassland (see Appendix S1). We used a reputable field guide [21] and an encyclopedia of reference material [22]–[28] to list habitat types regularly utilized by each species according to our category system. This method places each bird along a spectrum of habitat specialization, where species with a low SI score are specialists and species with a relatively high score are habitat generalists. Such a species-specific SI is likely to be more ecologically relevant than a binary specialist/generalist classification [29]. For each site, we calculated the mean SI for all species detected, weighted by the total density of each species in the assemblage. The resulting Assemblage Specialization Index (ASI) for each site was analyzed as a response variable to test its dependence on the explanatory variables.

### Explanatory variables

We examined five potential explanatory variables for similarity: patch type, woodland extent, habitat subdivision, land-use intensity and *Manorina* density. Continuous variables were converted to factors, each with two, three or four group levels, as a requirement of the PERMDISP test used in the statistical analysis (Table 2).

**Table 2:** Factors used in the PERMDISP test for homogeneity of dispersions for taxonomic and functional similarity between factor groups.

Factor	Group definitions
*mancol*	A, *Manorina* colonies absent from site with <2.5 average density of either *Manorina* species; P, *Manorina* colony present with ≥2.5 average density of either *Manorina* species.
*patch*	Ri: riparian or gallery woodland vegetation bordering watercourses; La: a large woodland patch ≥30 ha; Sm: a small woodland patch <30 ha; Li: a linear woodland patch greater than twice the width in length bordering roadsides and fence lines.
*extent10*	L, landscapes with ≤ 10% woodland cover; H, landscapes with > 10% woodland cover.
*extent15*	L, landscapes with ≤ 15% woodland cover; H, landscapes with > 15% woodland cover.
*extent20*	L, landscapes with ≤ 20% woodland cover; H, landscapes with > 20% woodland cover.
*extent25*	L, landscapes with ≤ 25% woodland cover; H, landscapes with > 25% woodland cover.
*extent30*	L, landscapes with ≤ 30% woodland cover; H, landscapes with > 30% woodland cover.
*subdivision3*	L, landscapes with ≤3 woodland patches; H, landscapes with >3 woodland patches.
*subdivision4*	L, landscapes with ≤4 woodland patches; H, landscapes with >4 woodland patches.
*subdivision5*	L, landscapes with ≤5 woodland patches; H, landscapes with >5 woodland patches.
*subdivision6*	L: landscapes with ≤6 woodland patches; H; landscapes with >6 woodland patches.
*subdivision7*	L, landscapes with ≤7 woodland patches; H, landscapes with >7 woodland patches.
*intensity*	P, pastoral land-use has the greatest extent within the matrix; D, dryland cropping has the greatest extent within the matrix; I, irrigated cropping has the greatest extent within the matrix.

We defined sites as being within colonies (colony present) and actively defended by *Manorina* honeyeaters as those sites having an average density per survey of ≥2.5 individuals/site for either *Manorina* species. We excluded sites (colony absent) with lesser densities because sites with only one or two individuals, such as individuals undergoing breeding dispersal or extra-territorial foraging forays, are unlikely to be within the actual territory area. With interspecific aggression directed towards birds within the colony area [30], *Manorina* honeyeaters outside their territory area may not have an influence on avian assemblages.

We quantified landscape structure within 1 km radius landscapes surrounding each site. The extent of woodland vegetation and different land-use cover types was mapped by visual interpretation in ArcMap 9.3 from Spot-5 multi-spectral satellite imagery [31]. The extent of vegetation was converted to a two level factor based on the percentage cover of woodland vegetation across the landscape, excluding the study patch, and ranged from 3% to 70% (mean: 20%, median: 17%). Because birds often exhibit a threshold response to landscape structure [32], [33], we created a total of five factors for woodland extent each with a different cut-off for low woodland extent including; 10%, 15%, 20%, 25 and 30% woodland vegetation cover.

Habitat subdivision was quantified as the number of woodland patches, excluding the study patch. This ranged between 1–22 (mean: 7, median: 6), and was also converted to a two level factor. We created five factors for habitat subdivision each with a different cut-off for low subdivision including three, four, five, six and seven woodland patches. Landscapes with greater woodland habitat subdivision had smaller woodland patch sizes, smaller mean distance between woodland patches and a greater amount of woodland-agricultural edge.

Land cover within the production matrix was mapped as either pasture, dryland cropping or irrigated cropping. A three level factor was used to categorize land-use intensity for each landscape by the dominant land-use type within the production matrix. We considered pastoral land-use to be low intensity because the required inputs are low and edge contrast with woodland patches is relatively low. Dryland cropping was considered intermediate intensity because inputs and outputs are moderate and habitat structure contrasts greatly with woodland. Irrigated land-use has relatively large inputs and outputs and was defined as high intensity.

### Statistical analysis

Multidimensional scaling (MDS) configuration plots were used to visualize patterns of similarity and the direction of differences in mean distance to group centroid, by superimposing group labels. The plots were produced using MDS analysis of Bray-Curtis similarity matrices in the PRIMER 6 version 6.1.13 [34] program. We used the default setting of 25 restarts, a minimum stress of 0.01 and Kruskal stress formula set to 1.

We then conducted multivariate analysis of differences in assemblage similarity using the PERMDISP routine in the PERMANOVA+ version 1.0.3 [34] extension. PERMDISP compares variation in measures of assemblage similarity between groups, with low dispersion indicating more homogeneous assemblages across sites within that group whereas groups with more variable assemblage similarity measures have more heterogeneous avian assemblages, with greater β diversity. PERMDISP has previously been used to analyze various biotic communities such as vegetation communities [35], soil fungal communities [36], soil seed-bank communities [37], and marine benthic communities [38], [39]; although the method appears to have not been used for the analysis of biotic homogenization of avian communities. PERMDISP tests the homogeneity of multivariate dispersions within factor groups based on deviations from the group centroid. This test uses the ANOVA F statistic to compare the distances from observations to their group centroid and therefore cannot test the effects of continuous variables, only factors. P-values are obtained from permutations of residuals, using permutations of samples among groups after centering all groups onto a common location. This removes any location differences and makes the obtained residuals exchangeable under the null hypothesis of homogeneity of dispersions, as opposed to location effects [40]. PERMANOVA performs these tests using a large random sample of F-statistics, recalculated for each randomized permutation. Under this method the probability of Type I error remains equal to the *a priori* chosen significance level despite multiple tests [41]. The PERMDISP routine is limited to testing a single factor at one time and cannot test the significance of interactions.

For factors found to be significant, we tested potential interactions with other explanatory variables in the R program [42]. Log transformed distances to group centroids generated from the PERMDISP routine for a significant factor were used as the response variable in a generalized linear model. We built separate models testing for an interaction between the main grouping factor identified in PERMDISP and the other explanatory variables (patch type, the extent of woodland (ha), the number of woodland patches and land-use intensity).

For each factor we also tested for an association with variation in site ASI with a Kruskal-Wallis test. We chose this non-parametric test due to unequal sample sizes and uneven variance between factor groups with non-normal distributions. A significant result for this test indicates a relationship between explanatory factors and habitat specialization across the avian assemblage.

These data were collected with permission from the University of Queensland Animal Ethics Committee (reference no. 811108) and a scientific purposes (non-protected areas) permit (permit no. WISP05443008) issued by the Queensland government Environmental Protection Agency under legislation S12(E) Nature Conservation (Administration) Regulation 2006. Private land was accessed after permission was granted by land owners/managers.

## Results

### Taxonomic homogenization

**Figure 2:**
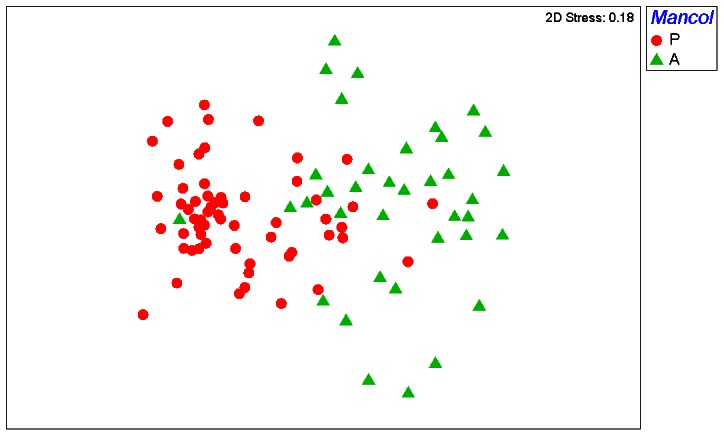
Non-metric multi-dimensional scaling graph produced from Bray Curtis taxonomic similarity of 96 sites using bird, log(x+1) transformed species density data. Solid red circles are sites colonized by either *Manorina* species (average density of *M. melanocephala* or *M. flavigula* ≥2.5), solid green triangles are sites where colonies are absent. The graph demonstrates a smaller dispersion of sites (taxonomic homogenization) where *Manorina* colonies are present compared to sites where colonies are absent.

We found a highly significant difference (p<0.01) in dispersions between sites with a *Manorina* colony present and those without (Table 3), with lower within-group dispersion for colonized sites (Figure 2). Group dispersions did not differ significantly with patch type or landscape structure. The explanatory factor *Manorina* colony was also significantly associated with variation in the assemblage similarity index (ASI), as indicated by the Kruskal-Wallis test results. There were no significant associations between variation in ASI and patch type or landscape structure. There were no statistically significant interactive effects between *Manorina* colony and any of the landscape structure variables on group dispersions (see Appendix S2).

**Table 3:** PERMDISP tests of homogeneity of dispersions (taxonomic similarity) results based on mean distance to group centroid for all groups within each factor, using log(x+1) transformed species density data.

	PERMDISP test			Kruskal-Wallis test		
Factor	F	df	p-value	chi-squared	df	p-value
*mancol*	11.53	1	<0.01	2.86	1	<0.01
*patch*	2.19	3	0.14	8.29	3	0.41
*extent10*	1.29	1	0.33	2.41	1	0.12
*extent15*	2.28	1	0.17	1.85	1	0.17
*extent20*	0.29	1	0.63	0.32	1	0.57
*extent25*	0.49	1	0.53	0.06	1	0.80
*extent30*	0.14	1	0.75	0.27	1	0.60
*subdiv3*	0.63	1	0.50	0.19	1	0.67
*subdiv4*	0.35	1	0.60	0.23	1	0.63
*subdiv5*	0.32	1	0.61	1.15	1	0.28
*subdiv6*	0.10	1	0.78	1.17	1	0.28
*subdiv7*	0.03	1	0.87	2.82	1	0.09
*intensity*	0.41	1	0.72	1.04	2	0.59

P-values obtained from permutations of residuals. Significant results indicate spatial taxonomic homogenization in relation to particular factors. In addition, Kruskal-Wallis one-way analysis of variance of median group ASI for each factor. Statistically significant results reject the null hypothesis of no difference between median group ASI.

### Functional homogenization

**Figure 3:**
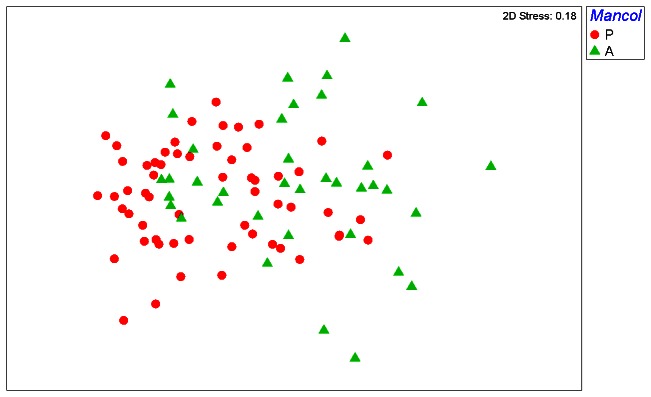
Non-metric multi-dimensional scaling graph produced from Bray Curtis functional similarity of 96 sites using bird, log(x+1) transformed functional group density data. Solid red circles are sites colonized by either *Manorina* species (average density of *M. melanocephala* or *M. flavigula* ≥2.5), solid green triangles are sites where colonies are absent. The graph demonstrates a smaller dispersion of sites (functional homogenization) where *Manorina* colonies are present compared to sites where colonies are absent.

**Figure 4:**
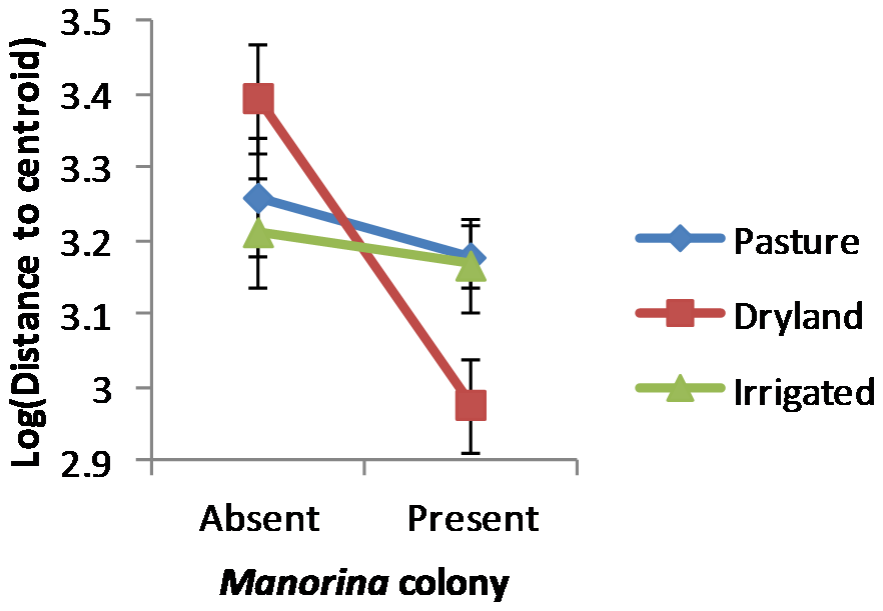
Plot of the mean log (Distance to centroid)±standard error, displaying the interaction between the presence of *Manorina* colonies and the intensity of land use in the surrounding landscape.

We found a highly significant difference (p<0.01) of dispersions based on functional group data between sites with a *Manorina* colony present and those without (Table 4), with lower within-group dispersion for colonized sites (Figure 3). The relationship between *mancol* and ASI was also highly significant (p<0.01), as indicated by the Kruskal-Wallis test results. Group dispersions did not differ significantly with the main effects of patch type or landscape structure, although one aspect of landscape structure did interact with the factor *Manorina* colony. There were no significant associations between variation in ASI and patch type or landscape structure. There was a statistically significant (p<0.05) interaction between *Manorina* colony and land-use intensity on group dispersions (Table 5). This interaction revealed that the significant reduction in within-group dispersion resulting from the presence of *Manorina* colonies was primarily associated with landscapes dominated by dryland cropping (Figure 4). No other interactions were statistically significant (see Appendix S3).

**Table 4:** PERMDISP tests of homogeneity of dispersions (functional similarity) results based on mean distance to group centroid for all groups within each factor, using log(x+1) transformed functional group density data.

	PERMDISP test			Kruskal-Wallis test		
Factor	F	df	p-value	chi-squared	df	p-value
*mancol*	7.59	1	<0.01	2.86	1	<0.01
*patch*	1.37	3	0.31	8.29	3	0.41
*extent10*	0.02	1	0.90	2.41	1	0.12
*extent15*	2.05	1	0.18	1.85	1	0.17
*extent20*	0.22	1	0.66	0.32	1	0.57
*extent25*	0.32	1	0.60	0.06	1	0.80
*extent30*	0.04	1	0.85	0.27	1	0.60
*subdiv3*	0.15	1	0.72	0.19	1	0.67
*subdiv4*	0.25	1	0.64	0.23	1	0.63
*subdiv5*	0.44	1	0.53	1.15	1	0.28
*subdiv6*	0.07	1	0.79	1.17	1	0.28
*subdiv7*	0.08	1	0.78	2.82	1	0.09
*intensity*	0.25	1	0.80	1.04	2	0.59

P-values obtained from permutations of residuals. Significant results indicate spatial functional homogenization in relation to particular factors. In addition, Kruskal-Wallis one-way analysis of variance of median group ASI for each factor. Statistically significant results reject the null hypothesis of no difference between median group ASI.

**Table 5:** Summary of the generalised linear model (glm) used to test the statistical significance of the interactive effect of *Manorina* colony and land-use intensity on group dispersions based in functional group density data.

Term	Estimate	Std. Error	t value	Pr(>|t|)
*Intercept*	3.40	0.08	44.75	<0.001
*mancol*	–0.42	0.11	–3.90	<0.001
*intensity*	–0.18	0.10	–1.80	0.075
*mancol:intensity*	0.37	0.15	2.52	<0.05
*Residual deviance*	5.70			
*Df*	90			

## Discussion

Our study contributes to the understanding of the process of land-use induced biotic homogenization and demonstrates the importance of interspecific interactions between locally native species in human-modified landscapes. Homogenization of avian communities has been documented in Europe [4], [10], [43], North America [44] and Africa [45], but tests conducted in Australia are rare [46]. We asked the question: can landscape structure and interspecific competition from native species, with static distribution ranges, drive biotic homogenization across space? Specifically, we found that interspecific competition from *Manorina* honeyeaters, rather than landscape structure, was the most significant driver of both taxonomic and functional homogenization of avian assemblages within woodlands fragmented by agriculture. The interaction with land-use intensity indicates that functional homogenization was greatest in landscapes dominated by dryland cropping, an intermediate-intensity land use.

### Pattern and process of biotic homogenization

In our study system, both taxonomic and functional homogenization resulted from the presence of colonies of two species of the *Manorina* genus, either the noisy miner or the yellow-throated miner (Figures 2 and 4). *Manorina* honeyeaters are gregarious birds, with coalitions of up to 50 birds aggressively defending territories with alarm calls, threat displays and direct attacks, and on occasion intruders are injured or even killed [30]. The noisy miner is particularly aggressive and its impact on avian assemblage structure is well documented [15], [17], [47], although it has only recently been shown to influence assemblage similarity [46]. At 63 g body weight, this species effectively excludes smaller (<50 g) avian species from its territories [13]. The yellow-throated miner also excludes smaller heterospecifics [48].

We found that as well as reducing species richness, both *Manorina* species also homogenize avian assemblages within woodland habitats. Although the decline in α diversity in response to *Manorina* species is well known, our results are novel because they demonstrate a more complex and independent process. Biotic homogenization is not synonymous with species invasion and extinction, and therefore cannot be assumed to covary predictably with species richness [3].

We suggest that *Manorina* honeyeaters are continuing to promote biotic similarity within remaining habitat in agricultural landscapes through their competitive interactions with other avian species. Some authors suggest the noisy miner has increased in density within its original range [17] in response to land clearance [49], habitat fragmentation [50], reduced habitat complexity [51], and grazing by livestock [52]. Both *Manorina* species have shown >20% increases in density across many regions in the last 30 years [53]. As these species increase in abundance, they are likely to occupy more sites across the landscape. Although our results quantify biotic homogenization across space, not time, the effect from *Manorina* honeyeaters on assemblage composition is likely to increase as more sites are occupied and colonies increase in size.

Although landscape change is thought to promote the abundance of *Manorina* honeyeaters in Australian agricultural landscapes, we found no statistically significant direct effects of landscape structure *per se* on the biotic homogenization of avian assemblages. This result is surprising and contrasts with previous studies that have found significant effects of habitat loss [54], habitat fragmentation [55] and land-use intensity [56] on the similarity of avian assemblages in agricultural landscapes. This study detected no influence of landscape structure despite examining multiple potential threshold points in the relationship between homogenization and both habitat extent and habitat subdivision.

Landscape structure was, however, an important influence on the extent to which *Manorina* colonies caused functional homogenization of avian assemblages. Our results suggest that functional homogenization due to *Manorina* honeyeaters is greatest in landscapes dominated by dryland cropping (Figure 4).The homogenizing effect of these honeyeaters is less pronounced in landscapes dominated by pasture or irrigated cropping. This effect may be the result of a trade-off generated by interactions between habitat and competition [57]. For example, if dryland landscapes represent high quality habitat with greater productivity for *Manorina* honeyeaters, colony members may invest more energy into territory defense through greater aggression and an increased rate of intruder exclusion [58]. Dryland landscapes may provide high quality habitat for *Manorina* honeyeaters due to an increased availability of resources. For example, certain insects may be associated with dryland crops such as wheat or barley, but may not be highly available in landscapes dominated by pasture or irrigated crops such as cotton. An alternative explanation for the increased effect of *Manorina* colonies in dryland landscapes may be that the functional diversity of species between sites is greater in dryland landscapes, in the absence of *Manorina* honeyeaters. Greater functional diversity within dryland landscapes may be explained by an intermediate intensity of land use in comparison to landscapes dominated by pasture or irrigated cropping, as suggested by the intermediate disturbance hypothesis [59]. The rate of disturbance in intermediate land-use intensity or dryland cropping landscapes may be optimal for woodland birds. Identifying the causal mechanisms responsible for the relationship between low functional similarity and intermediate land-use intensity in agricultural landscapes is a research priority and warrants further research.

Several studies have found strong relationships between the ratio of habitat specialists to generalists and biotic homogenization [12], [60]. Other authors have even defined functional homogenization using communitywide indices of habitat specialization [10], [29]. Previous studies suggest that changes to landscape structure such as habitat loss and fragmentation homogenize biotic assemblages due to adverse and disproportionate impacts on specialist species [10], [12], [54]. Habitat specialists are often the most affected by habitat changes in agricultural landscapes because they cannot utilize resources within the production matrix and the high edge contrast between the open area and natural habitats creates a barrier to their movements [61]–[63].

Our communitywide index of habitat specialization (ASI) also showed strong correlations with an environmental factor responsible for both taxonomic and functional assemblage similarity. Habitat specialization was lower where the presence of *Manorina* colonies had increased assemblage similarity, suggesting that both taxonomic and functional homogenization of avian assemblages within our study area results from the decline of habitat specialist species at the site-scale. In contrast, change in landscape structure was not correlated with variation in ASI, and this may explain why we found no direct effects of landscape structure on assemblage similarity.

### Implications for conservation

This study adds to the current understanding of the processes affecting avian assemblages in remnant habitat. It has identified interspecific competition from native species as a driving force in the replacement of specialist species with habitat generalists leading to biotic homogenization. Several *Manorina* honeyeaters, particularly the noisy miner, have benefitted from landscape modification for agricultural land-use [17], [49]–[51]. The homogenizing effect of these aggressive species on functional diversity also interacted with land-use intensity. Just as species invasions have been shown to homogenize biotic assemblages through the replacement of specialists with generalists [1], this finding contributes to a growing awareness that landscape modification can disrupt the competitive dynamics [11] responsible for species composition assembly rules [64], and result in homogeneous avian assemblages with the loss of specialist species [1], [3], [65]. This has implications for conservation world-wide, with demand for agricultural production continuing to increase [66], the threat of land-use induced biotic homogenization will continue to intensify at a global scale [5], [67], even in the absence of introduced species.

## Supporting Information

Appendix S1 **List of avian species included in the analysis indicating functional group and habitat associations.**
(PDF)

Appendix S2 **Summary of generalised liner models (glm) used to test interactive effects of Manorina colony presence and other explanatory variables on within-group dispersions based on species composition similarity.**
(PDF)

Appendix S3 **Summary of generalised liner models (glm) used to test interactive effects of Manorina colony presence and other explanatory variables on within-group dispersions based on functional group composition similarity.**
(PDF)
