# Supplementary material for: Influence of Interspecific Competition and Landscape Structure on Spatial Homogenization of Avian Assemblages
Source: PLoS One. 2013 May 28;8(5):e65299. doi: 10.1371/journal.pone.0065299 (PMC3665551; doi:10.1371/journal.pone.0065299)
Supplement: Appendix S1 — List of avian species included in the analysis indicating functional group and habitat associations. (PDF) [file pone.0065299.s001.pdf]

Appendix S1. Avian species included in the analysis (Christidis and Boles 2008). Foraging strata: G, ground; S, shrub; B, branch; C, canopy; A, aerial, and primary diet: G, granivore; F, frugivore; I, insectivore; N, nectarivore; C, carnivore, used to create functional groups. Tick marks indicate habitat associations used to calculate species specific habitat Specialization Index (SI).

Species name	Common name	Foraging strata	Primary diet	airspace	grasslands	herbfields	heathlands	shrublands	mallee	savanna	open woodlands	closed woodlands	riparian associations	open forests	closed forests	monsoon forest	vine forest	temperate rainforests	sub-tropical rainforests	tropical rainforests	mangroves	wetlands	Specialization Index
Acanthagenys rufogularis	spiny-cheeked honeyeater	C	F				✓	✓	✓		✓	✓											5
Acanthiza apicalis	inland thornbill	S	I					✓	✓		✓	✓		✓	✓								6
Acanthiza chrysorrhoa	yellow-rumped thornbill	G	I		✓			✓			✓			✓									4
Acanthiza lineata	striated thornbill	C	I					✓	✓		✓	✓	✓	✓	✓			✓	✓	✓			10
Acanthiza nana	yellow thornbill	C	I					✓			✓	✓	✓	✓									5
Acanthiza pusilla	brown thornbill	S	I				✓	✓			✓	✓		✓	✓			✓	✓	✓			9
Acanthiza reguloides	buff-rumped thornbill	G	I								✓			✓									2
Acanthiza uropygialis	chestnut-rumped thornbill	S	I					✓	✓		✓												3
Accipiter cirrhocephalus	collared sparrowhawk	A	C				✓	✓	✓		✓	✓		✓	✓							✓	8
Accipiter fasciatus	brown goshawk	G	C					✓	✓		✓	✓	✓	✓	✓			✓	✓	✓	✓		11
Aphelocephala leucopsis	southern whiteface	G	I					✓			✓	✓											3
Aprosmictus erythropterus	red-winged parrot	C	G		✓						✓		✓	✓	✓								5
Aquila audax	wedge-tailed eagle	G	C		✓	✓	✓	✓	✓		✓	✓		✓	✓								9
Artamus cinereus	black-faced woodswallow	A	I	✓	✓			✓			✓		✓	✓									6
Artamus cyanopterus	dusky woodswallow	A	I	✓				✓			✓	✓		✓	✓			✓	✓	✓			9
Artamus leucorynchus	white-breasted woodswallow	A	I	✓				✓			✓			✓				✓	✓	✓		✓	8
Artamus personatus	masked woodswallow	A	I		✓			✓	✓		✓	✓		✓				✓	✓	✓	✓		10
Artamus superciliosus	white-browed woodswallow	A	I	✓				✓			✓			✓									4
Barnardius zonarius	eastern ringneck	G	G					✓	✓		✓		✓	✓									5
Cacatua galerita	sulphur-crested cockatoo	G	G		✓				✓		✓	✓	✓	✓	✓		✓	✓	✓	✓			11
Cacatua sanquinea	little corella	G	G		✓			✓		✓	✓		✓										5
Cacomantis falbelliformis	fan-tailed cuckoo	S	I								✓			✓	✓			✓	✓	✓			6

Cacomantis variolosus	brush cuckoo	C	I								✓		✓	✓	✓	✓					✓		6
Chalcites basalis	Horsefield's bronze-cuckoo	S	I				✓	✓			✓	✓		✓	✓								6
Chalcites osculans	black-eared cuckoo	S	I		✓		✓	✓	✓		✓												5
Chthonicola sagittata	speckled warbler	G	I								✓	✓		✓	✓								4
Cincloramphus cruralis	brown songlark	G	I		✓			✓		✓	✓			✓									5
Cincloramphus mathewsi	rufous songlark	G	I		✓			✓			✓			✓									4
Circus approximans	swamp harrier	A	C																		✓	✓	2
Circus assimilis	spotted harrier	A	C		✓	✓		✓			✓											✓	5
Cisticola exilis	golden-headed cisticola	S	I		✓		✓	✓			✓			✓				✓	✓	✓		✓	9
Climacteris picumnus	brown treecreeper	B	I								✓	✓		✓									3
Colluricincla harmonica	grey shrike-thrush	C	I					✓	✓				✓					✓	✓	✓			6
Columba livia	rock dove	G	G		✓						✓												2
Coracina maxima	ground cuckoo-shrike	G	I					✓			✓		✓	✓									4
Coracina novaehollandiae	black-faced cuckoo-shrike	C	I	✓				✓			✓	✓		✓	✓			✓	✓	✓			9
Coracina papuensis	white-bellied cuckoo-shrike	C	I					✓			✓	✓	✓	✓	✓			✓	✓	✓	✓		10
Corcorax melanorhamphos	white-winged chough	G	I					✓			✓	✓		✓	✓								5
Corvus coronoides	Australian raven	G	I		✓						✓	✓		✓	✓								5
Corvus orru	Torresian crow	G	I					✓			✓	✓	✓	✓	✓			✓	✓	✓			9
Coturnix pectoralis	stubble quail	G	G		✓	✓	✓	✓															4
Coturnix ypsilophora	brown quail	G	G		✓		✓	✓					✓										4
Cracticus nigrogularis	pied butcherbird	G	C		✓		✓	✓			✓	✓		✓	✓			✓	✓	✓	✓		11
Cracticus tibicen	Australian magpie	G	I		✓		✓	✓			✓	✓		✓	✓			✓	✓	✓			10
Cracticus torquatus	grey-butcherbird	G	C								✓	✓	✓	✓	✓			✓	✓	✓		✓	9
Dacelo novaeguineae	laughing kookaburra	G	C								✓	✓		✓									3
Daphoenositta chrysoptera	varied sittella	B	I								✓	✓		✓	✓								4
Dicaeum hirundinaceum	mistletoebird	C	F					✓			✓	✓		✓	✓			✓	✓	✓	✓		9
Dromaius novaehollandiae	emu	G	F		✓		✓	✓	✓		✓	✓		✓	✓								8
Elanus axillaris	black-shouldered kite	G	C		✓		✓	✓	✓		✓												5

Entomyzon cyanotis	blue-faced honeyeater	C	N				✓				✓			✓		✓					✓		5
Eolophus roseicapillus	galah	G	G		✓			✓		✓	✓	✓	✓	✓									7
Eopsaltria australis	eastern yellow robin	G	I								✓	✓		✓	✓								4
Eurystomus orientalis	dollarbird	A	I								✓			✓	✓			✓	✓	✓			6
Falco berigora	brown falcon	G	C		✓	✓		✓	✓		✓	✓		✓									7
Falco cenchroides	nankeen kestrel	G	I		✓	✓	✓	✓	✓		✓	✓		✓									8
Falco longipennis	little falcon	A	C					✓			✓		✓	✓								✓	5
Falco subniger	black falcon	A	C		✓	✓		✓			✓	✓	✓	✓	✓							✓	9
Geopelia cuneata	diamond dove	G	G		✓					✓	✓												3
Geopelia humeralis	bar-shouldered dove	G	G				✓	✓		✓	✓	✓	✓	✓	✓						✓	✓	10
Geopelia striata	peaceful dove	G	G					✓			✓	✓		✓									4
Gerygone albogularis	white-throated gerygone	C	I					✓			✓	✓		✓	✓	✓					✓		7
Gerygone fusca	western gerygone	S	I					✓	✓		✓	✓	✓	✓	✓							✓	8
Glossopsitta concinna	musk lorikeet	C	N								✓			✓									2
Grallina cyanoleuca	magpie-lark	G	G		✓					✓	✓		✓	✓								✓	6
Grantiella picta	painted honeyeater	C	F								✓			✓									2
Haliaaeetus leucogaster	white-bellied sea-eagle	A	C		✓																	✓	2
Haliastur sphenurus	whistling kite	G	C		✓		✓	✓	✓		✓			✓									6
Hieraaetus morphnoides	little eagle	G	C					✓	✓		✓	✓	✓	✓	✓								7
Hirundapus caudacutus	white-throated needletail	A	I	✓							✓			✓				✓	✓	✓			6
Hirundo neoxena	welcome swallow	A	I	✓	✓		✓				✓	✓		✓									6
Lalage sueurii	white-winged triller	C	I		✓		✓	✓			✓		✓	✓	✓	✓					✓		9
Lichenostomus penicillatus	white-plumed honeyeater	C	N								✓		✓	✓									3
Lichenostomus virescens	singing honeyeater	S	N					✓			✓												2
Lichmera indistincta	brown honeyeater	S	N								✓	✓		✓	✓						✓		5
Lophoictinia isura	square-tailed kite	C	C		✓		✓	✓			✓		✓	✓	✓			✓	✓	✓			10
Malurus cyaneus	superb fairy-wren	G	I								✓	✓		✓	✓			✓	✓	✓			7
Malurus lamberti	variegated fairy-wren	G	I				✓	✓	✓		✓	✓											5

Malurus leucopterus	white-winged fairy-wren	G	I		✓		✓	✓															3
Malurus splendens	splendid fairy-wren	G	I					✓	✓		✓	✓											4
Melanodryas cucullata	hooded robin	G	I					✓	✓		✓			✓									4
Melithreptus brevirostris	brown-headed honeyeater	C	N								✓			✓									2
Melithreptus gularis	black-chinned honeyeater	C	N								✓			✓									2
Melopsittacus undulatus	budgerigar	G	G		✓			✓	✓		✓												4
Merops ornatus	rainbow bee-eater	A	I		✓			✓	✓		✓		✓	✓	✓	✓						✓	9
Microeca fascinans	jacky winter	G	I					✓			✓		✓	✓									4
Milvus migrans	black kite	G	C		✓			✓			✓		✓										4
Myiagra inquieta	restless flycatcher	G	I								✓		✓										2
Myiagra rubecula	leaden flycatcher	A	I								✓	✓	✓	✓				✓	✓	✓	✓		8
Neochmia modesta	plum-headed finch	G	G		✓						✓			✓									3
Neophema pulchella	turquoise parrot	G	G							✓	✓		✓	✓									4
Northiella haematogaster	blue bonnet	G	G		✓			✓	✓		✓												4
Nymphicus hollandicus	cockatiel	G	G		✓			✓	✓	✓	✓		✓	✓									7
Ocyphaps lophotes	crested pigeon	G	G		✓			✓		✓	✓		✓										5
Oreoica gutturalis	crested bellbird	G	I		✓			✓	✓		✓	✓											5
Oriolus sagittatus	olive-backed oriole	C	I								✓		✓	✓				✓	✓	✓			6
Pachycephala pectoralis	golden whistler	S	I					✓	✓				✓				✓	✓					5
Pachycephala rufiventris	rufous whistler	C	I					✓			✓	✓	✓	✓	✓			✓	✓	✓	✓		10
Pardalotus punctatus	spotted pardalote	C	I								✓	✓		✓	✓								4
Pardalotus striatus	striated pardalote	C	I					✓			✓	✓		✓	✓								5
Petrochelidon ariel	fairy martin	A	I	✓	✓			✓			✓	✓										✓	6
Petrochelidon nigricans	tree martin	A	I	✓	✓			✓			✓	✓		✓	✓							✓	8
Petroica boodang	scarlet robin	G	I						✓		✓	✓		✓	✓								5
Petroica goodenovii	red-capped robin	G	I					✓			✓	✓											3
Phaps chalcoptera	common bronzewing	G	G				✓	✓	✓	✓	✓	✓		✓									7
Philemon citreogularis	little friarbird	C	N				✓				✓			✓							✓	✓	5

Philemon corniculatus	noisy friarbird	C	N				✓	✓			✓	✓		✓	✓							✓	7
Platycercus adscitus	pale-headed rosella	G	G		✓						✓			✓									3
Platycercus eximius	eastern rosella	G	G		✓						✓												2
Plectorhyncha lanceolata	striped honeyeater	C	N						✓		✓	✓	✓										4
Pomatostomus temporalis	grey-crowned babbler	G	I					✓		✓	✓		✓	✓									5
Psephotus haematonotus	red-rumped parrot	G	G		✓						✓												2
Ptilonorhynchus maculatus	spotted bowerbird	S	F								✓	✓											2
Rhipidura albiscapa	grey fantail	C	I				✓	✓	✓		✓	✓	✓	✓	✓			✓	✓	✓			11
Rhipidura leucophrys	willie wagtail	G	I		✓						✓			✓									3
Scythrops novaehollandiae	channel-billed cuckoo	C	F								✓	✓		✓	✓				✓	✓			6
Smicrornis brevirostris	weebill	C	I					✓	✓		✓	✓		✓	✓								6
Stagonopleura guttata	diamond firetail	G	G		✓				✓		✓			✓									4
Strurnus tristis	common myna	G	I		✓			✓	✓	✓	✓		✓	✓							✓	✓	9
Struthidea cinerea	apostlebird	G	G					✓			✓	✓		✓	✓								5
Sturnus vulgaris	common starling	G	G		✓	✓	✓	✓	✓		✓			✓									7
Sugomel niger	black honeyeater	S	N					✓			✓												2
Taeniopygia bichenovii	double-barred finch	G	G		✓			✓			✓	✓		✓				✓	✓	✓	✓		9
Taeniopygia guttata	zebra finch	G	G	✓				✓	✓		✓												4
Todiramphus sanctus	sacred kingfisher	G	I								✓			✓		✓	✓	✓					5
Trichoglossus chlorolepidotus	scaly-breasted lorikeet	C	N				✓				✓	✓		✓								✓	5
Trichoglossus haematodus	rainbow lorikeet	C	N				✓				✓	✓		✓	✓	✓				✓			7
Zosterops lateralis	silvereye	C	I				✓	✓			✓	✓		✓	✓			✓	✓	✓	✓		10
