# Supplementary material for: Influence of Interspecific Competition and Landscape Structure on Spatial Homogenization of Avian Assemblages
Source: PLoS One. 2013 May 28;8(5):e65299. doi: 10.1371/journal.pone.0065299 (PMC3665551; doi:10.1371/journal.pone.0065299)
Supplement: Appendix S2 — Summary of generalised liner models (glm) used to test interactive effects of Manorina colony presence and other explanatory variables on within-group dispersions based on species composition similarity. (PDF) [file pone.0065299.s002.pdf]

Appendix S2. Summary of generalised liner models (glm) used to test interactive effects of Manorina colony presence and other explanatory variables on within-group dispersions based on species composition similarity.

Model: log(Distance) ~ mancol*extent

Term	Estimate	Std. Error	t value	Pr(>|t|)
Intercept	3.78	0.03	120.96	<0.001
mancol	-0.14	0.04	-3.56	<0.001
extent	-0.01	0.03	-0.51	0.610
mancol:extent	-0.01	0.04	-0.17	0.870
Residual deviance	36.32			
Df	92			

Model: log(Distance) ~ mancol*subdivision

Term	Estimate	Std. Error	t value	Pr(>|t|)
Intercept	3.78	0.03	119.01	<0.001
mancol	-0.14	0.04	-3.43	<0.001
subdivision	-0.01	0.04	-0.16	0.871
mancol:subdivision	-0.01	0.04	-0.31	0.753
Residual deviance	3.32			
Df	92			

Model: log(Distance) ~ mancol*patch

Term	Estimate	Std. Error	t value	Pr(>|t|)
Intercept	3.73	0.62	59.51	<0.001
mancol	-0.09	0.08	-1.18	0.240
patch	0.15	0.09	1.74	0.085
mancol:patch	-0.17	0.11	-1.55	0.125
Residual deviance	3.11			
Df	88			

Model: log(Distance) ~ mancol*intensity

Term	Estimate	Std. Error	t value	Pr(>|t|)
Intercept	3.81	0.06	66.73	<0.001
mancol	-0.16	0.08	-1.96	0.054
intensity	-0.09	0.08	-1.20	0.896
mancol:intensity	0.01	0.11	0.09	0.928
Residual deviance	3.23			
Df	90			
