# Supplementary material for: Influence of Interspecific Competition and Landscape Structure on Spatial Homogenization of Avian Assemblages
Source: PLoS One. 2013 May 28;8(5):e65299. doi: 10.1371/journal.pone.0065299 (PMC3665551; doi:10.1371/journal.pone.0065299)
Supplement: Appendix S3 — Summary of generalised liner models (glm) used to test interactive effects of Manorina colony presence and other explanatory variables on within-group dispersions based on functional group composition similarity. (PDF) [file pone.0065299.s003.pdf]

Appendix S3. Summary of generalised liner models (glm) used to test interactive effects of Manorina colony presence and other explanatory variables on within-group dispersions based on functional group composition similarity.

Model: log(Distance) ~ mancol*extent

Term	Estimate	Std. Error	t value	Pr(>|t|)
Intercept	3.28	0.04	78.06	<0.001
mancol	-0.15	0.05	-2.71	<0.01
extent	-0.08	0.04	-1.93	0.060
mancol:extent	0.08	0.05	1.55	0.125
Residual deviance	6.02			
Df	92			

Model: log(Distance) ~ mancol*subdivision

Term	Estimate	Std. Error	t value	Pr(>|t|)
Intercept	3.28	0.04	75.60	<0.001
mancol	-0.15	0.06	-2.63	<0.01
subdivision	0.00	0.05	-0.09	0.920
mancol:subdivision	0.03	0.06	0.51	0.610
Residual deviance	6.22			
Df	92			

Model: log(Distance) ~ mancol*patch

Term	Estimate	Std. Error	t value	Pr(>|t|)
Intercept	3.26	0.09	38.06	<0.001
mancol	-0.03	0.11	-0.29	0.772
patch	0.05	0.12	0.43	0.668
mancol:patch	-0.21	0.15	-1.36	0.179
Residual deviance	5.81			
Df	88			

Model: log(Distance) ~ mancol*intensity

Term	Estimate	Std. Error	t value	Pr(>|t|)
Intercept	3.40	0.08	44.75	<0.001
mancol	-0.42	0.11	-3.90	<0.001
intensity	-0.18	0.10	-1.80	0.075
mancol:intensity	0.37	0.15	2.52	<0.05
Residual deviance	5.70			
Df	90			
